# Predicting Patient-Specific Tumor Dynamics: How Many Measurements Are Necessary?

**DOI:** 10.3390/cancers15051368

**Published:** 2023-02-21

**Authors:** Isha Harshe, Heiko Enderling, Renee Brady-Nicholls

**Affiliations:** 1 Department of Integrated Mathematical Oncology, H. Lee Moffitt Cancer Center & Research Institute, Tampa, FL 33612, USA; 2 Department of Cell, Molecular, and Microbiology, University of South Florida, Tampa, FL 33620, USA; 3 Department of Genitourinary Oncology, H. Lee Moffitt Cancer Center & Research Institute, Tampa, FL 33612, USA; 4 Department of Radiation Oncology, H. Lee Moffitt Cancer Center & Research Institute, Tampa, FL 33612, USA

**Keywords:** patient-specific, prediction, logistic growth

## Abstract

**Simple Summary:**

Accurately predicting tumor growth is an important component in effectively treating patients; unfortunately, acquiring sufficient data to correctly predict when a patient will progress on treatment often comes too late. In this study, we investigated the sufficient number of tumor volume measurements required to accurately predict logistic tumor growth. The model was calibrated to tumor volume data from 18 untreated breast cancer patients using a varying number of measurements. We found the number of data points necessary to be a function of the noise level and the acceptable error of the to-be-determined model parameters. This study will provide a metric by which clinicians can determine when sufficient data have been collected to confidently predict patient-specific growth dynamics, which will be aimed at assisting treatment decision-making.

**Abstract:**

Acquiring sufficient data is imperative to accurately predict tumor growth dynamics and effectively treat patients. The aim of this study was to investigate the number of volume measurements necessary to predict breast tumor growth dynamics using the logistic growth model. The model was calibrated to tumor volume data from 18 untreated breast cancer patients using a varying number of measurements interpolated at clinically relevant timepoints with different levels of noise (0–20%). Error-to-model parameters and the data were compared to determine the sufficient number of measurements needed to accurately determine growth dynamics. We found that without noise, three tumor volume measurements are necessary and sufficient to estimate patient-specific model parameters. More measurements were required as the level of noise increased. Estimating the tumor growth dynamics was shown to depend on the tumor growth rate, clinical noise level, and acceptable error of the to-be-determined parameters. Understanding the relationship between these factors provides a metric by which clinicians can determine when sufficient data have been collected to confidently predict patient-specific tumor growth dynamics and recommend appropriate treatment options.

## 1. Introduction

Female breast cancer has the highest rate of cancer incidence in the world (11.7%), even surpassing lung cancer rates for both men and women combined (11.4%) [1]. Rates of female breast cancer incidence have been on the rise in recent years due to societal lifestyle changes, including lower fertility rates as women bear children at later ages and increased obesity rates [2]. In the United States, about one in eight women will receive a diagnosis of breast cancer in their lifetime, and breast cancer is the second leading cause of cancer deaths in females, even though survival rates have been on the rise [3,4]. Timely diagnosis and treatment are essential to breast cancer survival. The American Cancer Society recommends that women aged 40–54 years conduct mammography screenings annually to ensure early detection of breast cancer [3].

Whilst treatment guidelines have been established to effectively treat the cancer based on large clinical trials, how to tailor treatments to individual patients with comparable clinical features remains challenging. Clinical detection of a tumor offers only a static snapshot of the tumor growth history and future dynamics. Being able to accurately predict the growth dynamics of a patient’s tumor may offer a novel tool to contribute to integrated multidisciplinary decision-making. This is where mathematical models come into the arena—these models can take existing tumor measurements and predict how the tumor will grow [5]. One of the most commonly used models in mathematical oncology is the logistic growth model [5,6,7]. Unlike exponential growth with a constant volume doubling time, logistic growth decelerates as it approaches limitations to tumor expansion, called the carrying capacity. The carrying capacity is defined as the maximum tumor volume that can be sustained given the patient’s tumor biology, including immune status, tumor oxygenation, and the physical size of the breast. Two patients with identical tumor volumes can have vastly different growth dynamics based on their individual carrying capacities, and, thus, different responses to therapy [8,9,10].

The motivation to use the logistic growth model for breast cancer comes from preceding literature which promotes the utility of logistic growth as a means to predict tumor growth. Atuegwu et al. [5] used the logistic growth model to predict breast cancer patient responses to neoadjuvant chemotherapy using MRI data from before, during, and after treatment. Logistic growth has also been used for radiation therapy studies. Logistic growth and radiation response models were able to fit retrospective longitudinal non-small-cell lung cancer and head and neck cancer patient data across multiple institutions with high accuracy (R^2^ = 0.98). They defined the tumor-volume-to-carrying-capacity ratio as the proliferation saturation index (PSI), establishing it as the most important patient-specific parameter to determine treatment response [8,9,10]. The logistic growth model has not only been used with treatment data but also applied to untreated breast cancer patients. Spratt et al. fit observational breast cancer data for 448 patients to various mathematical models, including exponential, logistic, and Gompertzian growth. Each patient data set contained between two and six measurements and logistic growth best described the breast cancer growth dynamics [11].

In order to use tumor growth dynamics to help guide treatment decisions, we must be able to confidently derive the putative patient-specific carrying capacity. The aim of this study was to investigate the number of volume measurements necessary to predict tumor growth dynamics using the logistic growth model. The logistic model was calibrated to volumetric data from 18 untreated breast cancer patients. We found that without noise, three tumor volume measurements are necessary and sufficient to estimate patient-specific model parameters. More measurements were required as the level of noise increased. Estimating the tumor growth dynamics was shown to depend on the tumor growth rate, clinical noise level, and acceptable error of the to-be-determined parameters. To our knowledge, this is the first comprehensive analysis of the minimum number of measurements that are necessary to determine the growth rate and carrying capacity of a patient’s breast tumor. By conducting error analyses to determine how many measurements are necessary to predict patient dynamics given a certain set of conditions, we hope to strike a balance between reducing wait times until treatment and improving accuracy in predicting the patient’s tumor dynamics.

## 2. Methods

### 2.1. Clinical Data

The data used herein was derived from the 1979 study by Heuser et al. [12] of untreated breast cancer in 18 patients. Tumor dimensions were recorded at two time points to determine the tumor doubling time. Based on the given major and minor axes values for the tumor and the doubling times, we calculated the tumor radius and the tumor volume for each patient. The tumor radius was calculated by:

$$r=\frac{2a+b}{6},$$

where r is the radius, and a and b are the major and minor axes, respectively. With this value for the radius, the initial and second tumor volumes were calculated for three different shapes using the following equations:

Sphere     $V=\frac{4\pi r^{3}}{3},$Cylinder     $V=\pi r^{2}h,$Oblate Sphere     $V=\frac{4\pi a^{2}b}{3},$where V is the tumor volume, r is the tumor radius, h is the height of the cylinder, and a and b are the radii of the major and minor axes, respectively.

After calculating each patient’s initial ($V_{0}$) and final ($V_{t}$) tumor volumes for each shape, the doubling time (DT) was calculated using:

$$DT=\frac{\ln\left(2\right)}{y},$$

where $y=\frac{\ln\left(V_{t}\right)-\ln\left(V_{0}\right)}{t}.$ The doubling times for each of the three shapes were compared to the actual doubling time provided by Heuser et al. to determine the most accurate volume calculation for each patient. We found that for all of the patients, the cylinder volume calculations were the most accurate. Patients for whom doubling time was not listed were omitted from this study.

### 2.2. Mathematical Model

Tumor volume V dynamics were described using the logistic growth model given by the ordinary differential equation:

(1) $$\frac{dV}{dt}=\alpha V\left(1-\frac{V}{K}\right),$$

where α is the intrinsic, exponential tumor growth rate and K is the tumor carrying capacity.

**Figure 1:**
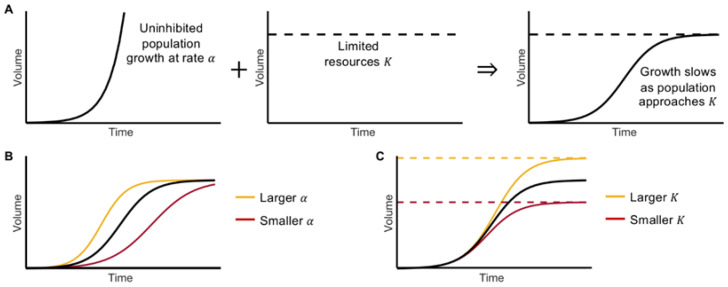
The Logistic Growth Model. (**A**) Volume grows uninhibited at rate α in the presence of unlimited resources. As the available resources decrease, growth slows and approaches the carrying capacity K. (**B**) Increasing (decreasing) the value of α allows the population to reach K faster (slower). (**C**) Increasing (decreasing) the value of K allows for the volume to grow larger (smaller).

Logistic growth takes on a sigmoidal, S-shaped curve. Initially, when the tumor volume is far from its carrying capacity, growth resembles exponential growth (see Figure 1A). In the presence of limited resources, the volume growth slows and approaches the carrying capacity of the population K. Mathematically, with increasing volume, the volume-to-carrying capacity ratio, $\frac{V}{K}$, increases and the net growth rate declines linearly until the volume approaches K. This growth can be characterized into four phases: initiation, acceleration, deceleration, and saturation [13]. The values of the growth rate α and carrying capacity K determine the model dynamics. A larger growth rate will result in the volume reaching its carrying capacity sooner, while a smaller α will increase the time it takes to reach K (see Figure 1B); similarly, a larger K (i.e., more resources) will allow for a larger end volume, whereas a smaller K has opposing effects (see Figure 1C).

### 2.3. Parameter Optimization

Parameter optimization [14] was used to determine the growth rate α and carrying capacity K to accurately describe the patient data. Nested optimization was used to simultaneously fit the model to the data from all 18 patients, whereby α was uniform among all patients and K was patient-specific. Given that each patient only had two volume measurements, the uniform α and patient-specific K values were used to simulate the model and obtain “ground truth” tumor volumes at different time steps along the individual growth curves for each patient. The curve was sampled at clinically relevant timepoints corresponding to typical radiological scan times prior to treatment. The initial point represents the volume at the first abnormal mammogram, which was taken directly from Heuser et al. [12]. The remaining sampled points occurred on days 7, 30, 60, 90, and 120, which may correspond to a follow-up screening, second opinion, and potential wait times until treatment [15,16,17].

Parameter optimization was used to fit the model to the data using three, four, five, and six measurements. The nominal carrying capacity to initialize the optimization procedure was set to be the last interpolated data point. That is, for three, four, five, and six measurements, the nominal carrying capacity would be the patient-specific tumor volume at days 30, 60, 90, and 120, respectively. Both the growth rate and the carrying capacity were optimized. For each patient, we recorded the optimizer-derived growth rate, the percent error to the original growth rate, the optimizer-derived carrying capacity, the percent error to the original carrying capacity, and the relative mean squared error to the original interpolated data points and the final tumor volume.

### 2.4. Adding Uncertainty (Noise) to the Data

It is generally accepted that clinical data contains noise, due to a combination of factors including, but not limited to, radiographic imaging resolution and physician contouring preference [18,19,20,21]; to account for this, we added noise to the data points interpolated from the original curves with noise levels of 5%, 10%, and 20%. Note, the initial and final tumor volume measurements were taken to be without error. To add noise, we used the following equations:

$$\begin{array}{c} \mathrm{noise}=a+\left(b-a\right)r,\\ V_{N}=V+\left(V\;\times \;\mathrm{level}\;\times \;\mathrm{noise}\right), \end{array}$$

where noise is the amount of uncertainty added to the data, with a=1,  b=−1,  r is a random number from 0 to 1, V is the original tumor volume, $V_{N}$ is the tumor volume with noise, and level is the noise level (either 0.05, 0.1, or 0.2 for respectively 5%, 10%, or 20%). We conducted 10 simulations per patient to account for the stochasticity in r.

### 2.5. Error Calculation and Comparison to Data

For each noise level, we determined the error between the optimization-derived parameter values and the original parameter values. The relative mean squared error to the interpolated data points is calculated using:

$$rMSE=\left(\frac{1}{N}\right)\overset{N}{\underset{i=1}{\sum }}{\left(V_{m}-V_{d}\right)}^{2},$$

where N is the number of interpolated data points (including the final tumor volume for each patient), $V_{m}$ is the tumor volume simulated by the model at each time point, and $V_{d}$ is the interpolated tumor volume at each time point. This was repeated across all the data sets with the different number of measurements and across all of the noise levels.

The Mann-Whitney U test was used to evaluate the comparisons between distributions of error values across the different numbers of measurements. This test was used because it compares the median ranks between paired data. Since all the error distributions were for the same patient set, the data could be considered paired. The comparisons were made for three versus four points, four versus five points, and five versus six points.

## 3. Results

### 3.1. Parameter Fits with Uniform Growth Rates Yield Patient-Specific Carrying Capacities

**Figure 2:**
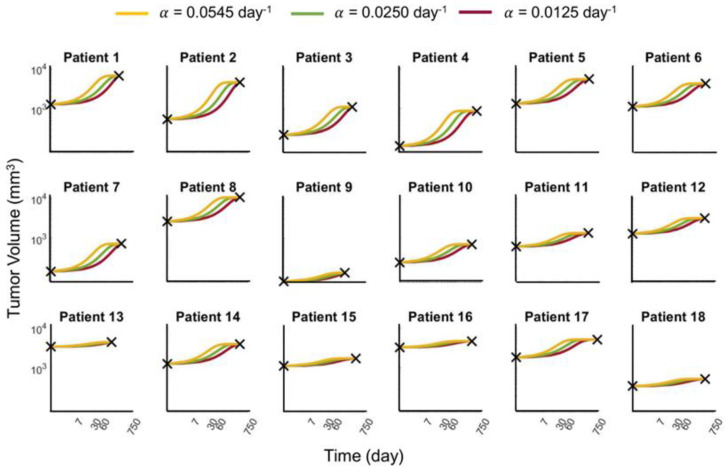
Logistic tumor growth curves of 18 untreated breast cancer patients. The black × markers denote the initial and final tumor volumes abstracted from clinical measurements. Three different curves were fitted through these two data points; each corresponds to the different growth rates of α = 0.0125 day^−1^, 0.025 day^−1^, and 0.0545 day^−1^ (red, green, and yellow curves, respectively) uniformly for all 18 patients. For easy comparison across the patient population, all curves are plotted on the same log-log scale with tumor volumes ranging from 80 to 11,000 mm^3^, and time ranging from zero to 750 days.

Using the logistic growth model, we first sought to determine the growth rate and carrying capacity values that could accurately describe patient-specific dynamics from 18 untreated breast cancer patients (see Methods). Given the limitation of only two available data points per patient, as well as the model being governed by just two model parameters, multiple growth rates could be used to fit the data equally well. We chose three growth rates (α∈0.0125, 0.025, and 0.0545 day^−1^) to fit the model and used parameter optimization [14] to find the corresponding carrying capacities for each patient individually (see Supplementary Figure S1). These curves (see Figure 2) and their associated growth rate and carrying capacity parameter values served as the “ground truth” upon which all further analyses were compared.

### 3.2. Perfect Data Yield Accurate Patient-Specific Carrying Capacity Estimates

**Figure 3:**
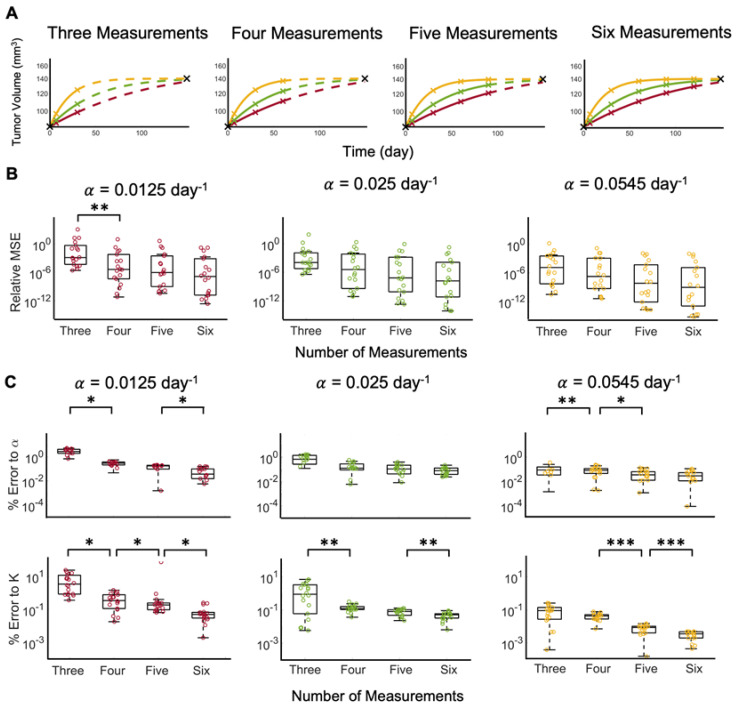
Tumor growth trajectory predictions from optimized model fits to ground truth data for representative patient 9. (**A**) Model fit to data using three, four, five, or six measurements (solid curve) to predict the tumor growth dynamics forward in time (dashed curve) for data generated with the different ground truth growth rates (α= 0.0125 (red), 0.025 (green), or 0.0545 day^−1^ (yellow)). The black markers are the original tumor volumes taken from Heuser et al., whereas the colored markers represent the interpolated tumor volumes at days 7, 30, 60, 90, and 120. Since the interpolated data points are specific to each growth rate, the color of the marker matches the color of the curve from which the point was interpolated. (**B**) Relative mean square error (MSE) between model fit and prediction and data generated as a function of number of measurements included in model fit, with the different ground truth growth rates, α. (**C**) Percent error of model estimated growth rates, α, and carrying capacities, *K*, as a function of the number of measurements included in model fit for the different ground truth growth rates. In **B**-**C**, statistical significance is denoted by asterisks (* for *p* < 0.01, ** for *p* < 0.005, and *** for *p* < 0.001).

Without noise in the clinical data and with a known growth rate α, the carrying capacity of Equation (1) can be estimated from the analytical solution to the logistic equation. If both model parameters are unknown, a priori, fitting the solution of Equation (1) to three ground truth data points at times 0, 7, and 30 days yields sufficient fits to the last data points (see Figure 3A). Increasing the number of measurements to four, five, and six does not noticeably further increase the fit to the last data points. A comparison of the relative mean squared error (MSE) shows that increasing the number of data points decreases the relative MSE, and the difference is statistically significant when comparing three versus four measurements for α=0.0125 (see Figure 3B). Further comparison of the error to the parameters shows that increasing the number of measurements significantly decreases the errors (see Figure 3C). Model fits to tumor volume dynamics with the slowest ground truth growth rate, α=0.0125 day^−1^, yields the highest average percent error to parameters α (5.056%) and K (0.851%). As the growth rate increases, the model reaches carrying capacity in a shorter time; therefore, at a larger growth rate, more of the dynamics of the curve are known in a shorter time period and, therefore, fewer measurements may be necessary.

### 3.3. Increasing Noise in the Data Requires Increasing the Number of Data Points to Estimate Patient-Specific Carrying Capacity

**Figure 4:**
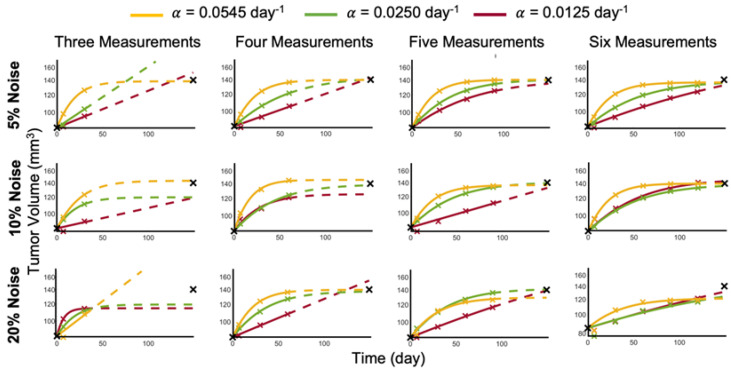
Logistic growth training (solid curves) and predictions (dashed curves) for Patient 9 with different percent noise in the training data.

To account for measurement error depending on the imaging method used [18,19,20,21], we added noise to the sampled data points and determined whether the model could accurately recapitulate the data or not. With as little as 5% noise added to the data, three measurements are no longer sufficient across all of the ground truth growth rates to train the logistic growth parameters to accurately predict the last data point. For data generated at the lower growth rates (α=0.0125, 0.025 day^−1^), the tumor volume at the last data point is estimated to be much higher than the patient data (see Figure 4). With the addition of a fourth measurement, the logistic model parameters can be correctly identified to accurately predict the tumor volumes at the last data point for all considered ground truth growth rates. Larger noise levels of 10% or 20% require additional data points for up to five and six measurements, respectively, to adequately forecast future tumor volumes (see Figure 4).

**Figure 5:**
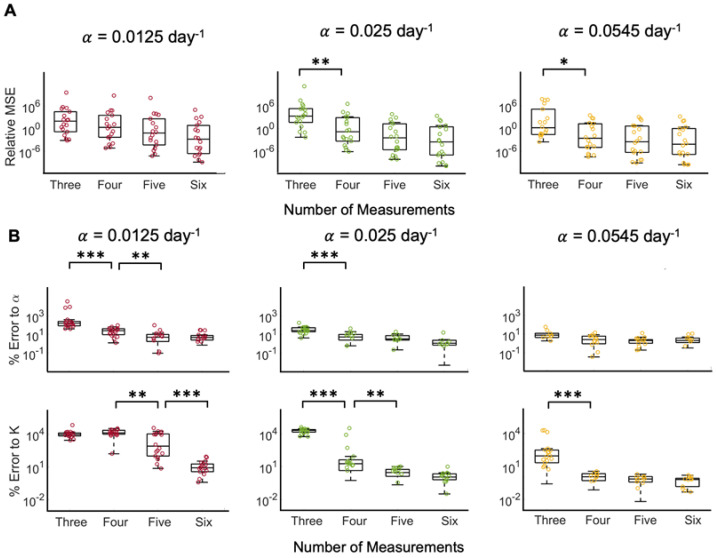
Error distributions of model fit to data with 5% noise. (**A**) Relative mean squared error (MSE) of model fit to ground truth data with different growth rates as a function of different measurements included in parameter estimation. (**B**) Percent error of model fit-derived logistic growth parameters (growth rate, α, and carrying capacity, *K*) to ground truth parameters as a function of different measurements included in parameter estimation. Statistical significance is denoted by asterisks (* for *p* < 0.01, ** for *p* < 0.005, and *** for *p* < 0.001).

For all noise levels (5% (see Figure 5); 10% (see Figure S2), 20% (see Supplementary Figure S3)), the relative MSE between model prediction and ground truth data reduces with additional measurements included in model fitting and parameter estimation. Regardless of which noise was added to the data, including four measurements instead of three significantly reduced the relative MSE for data generated with a high growth rate α=0.0545 day^−1^. Of interest, while additional measurements yielded visually improved fits, the improvement in fitting accuracy did not reach statistical significance. Analysis of the percent error of the estimated to the ground truth logistic growth model parameters, however, showed significant and clinically meaningful reductions in parameter errors with additional measurements (see Figure 5, and Supplementary Figures S2 and S3).

**Figure 6:**
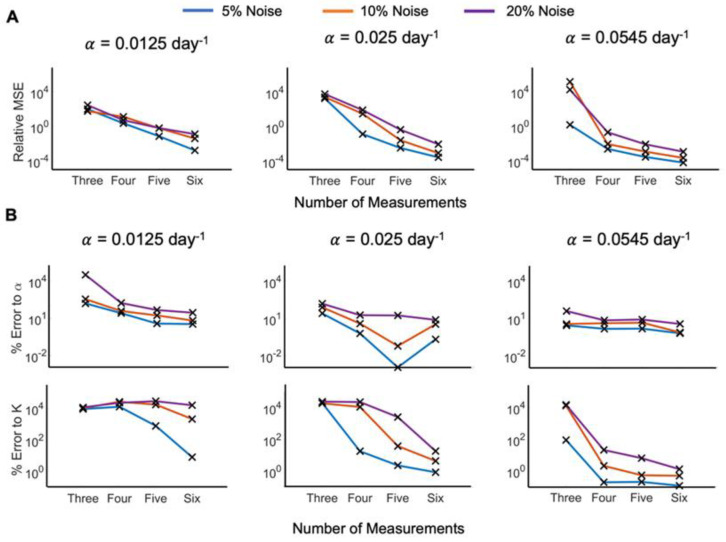
Comparison of error analyses for model fit and percent error across the different noise levels. (**A**) Relative mean squared error (MSE) between model prediction and ground truth data for different growth rates and noise levels in the data. (**B**) Comparison of percent error of model parameters (growth rate, α, and carrying capacity, *K*) to ground truth for different growth rates and noise levels in the data.

We have shown that the number of points necessary to accurately predict patient tumor growth, determined by the minimal error to the original data and parameters, is a function of the uniform growth rate, data noise level, and desired error range. The number of measurements necessary to predict a patient’s tumor growth may further depend on the clinician’s (un)certainty in data collection and acceptable error in model dynamics forecasts. From this, we can evaluate how many measurements should be taken to predict the patient’s tumor growth with acceptable relative MSE, or percent error to logistic growth rate, α, or carrying capacity, *K*. We evaluated the median values for the error distributions against the number of measurements used for the different noise levels (see Figure 6). Dependent on the clinicians’ accepted level of uncertainty, this can be used to determine how many measurements are sufficient to confidently predict responses.

## 4. Discussion

The aim of this study was to determine the minimum number of screenings that would be necessary in order to determine a cancer patient’s tumor dynamics using the logistic growth model. Using tumor volume data from untreated breast cancer patients, we fitted all of the patient trajectories to the logistic growth model and interpolated data at clinically relevant time points. Using these data, we were able to produce tumor growth dynamic curves and compare the use of three, four, five, and six measurements along the ground truth data. We added varying levels of noise to the interpolated data in order to capture a variety of possible patient scenarios.

Across growth rates and noise levels, we saw a trend where the median error decreases as the number of measurements increases. This is expected as having more measurements along the curve, especially further along the curve, provides more information into the dynamics of the logistic growth curve; however, the cost associated with having more measurements is time until treatment. This study aimed to provide the first insights into how many data points are necessary to estimate logistic growth parameters and predict tumor dynamics, dependent on uncertainty in clinically collected data and the clinician’s acceptable error in model prediction.

Across all of the noise levels, as the growth rate increases, the number of measurements necessary decreases. This could be due to the fact that at higher growth rates, the curves reach their carrying capacity in less time than at slower growth rates, and, thus, are able to capture more of the patient’s tumor dynamics in less time. At varying noise levels, we also found that as the noise level increased, the number of necessary measurements also increased to balance out the uncertainty in the data collected.

This is the first study, to our knowledge, that uses untreated patient-specific data to investigate when sufficient data have been collected to determine growth dynamics. Studies conducted in the past 10 years have primarily focused on predicting patient-specific responses to treatment to determine when and how to treat patients more effectively. While this is important, understanding untreated growth dynamics provides important information about the patient’s system unperturbed by treatment that oncologists might use to determine when to begin treatment or how best to administer it.

The presented study has focused on the logistic growth model and its two parameters, the growth rate and carrying capacity. If using an alternative model to predict tumor growth, for example, Gompertzian growth, the number of measurements necessary may vary, based on the parameters and dynamics of the model. When comparing models, determining the minimum number of measurements needed by each model for each distinct cancer type and patient cohort is likely a function of the specific model chosen, and the number of parameters being estimated. This will be explored in a future study.

It is also worth noting that tumor volume alone may not be fully indicative of a breast cancer patient’s condition. Additional prognosis factors such as the proliferation marker Ki67 or overexpression of HER2, as well as the mutational capacity of the tumor, might be useful in determining patient-specific carrying capacities. These should be considered in future studies; additionally, socio-economic factors might contribute to longer or shorter wait times and are, thus, important to consider when determining when sufficient data have been collected to determine patient-specific tumor growth dynamics.

## 5. Conclusions

In conclusion, this study found that the number of measurements necessary to predict the tumor growth dynamics for breast cancer patients is reliant upon multiple factors. These factors include the growth rate of the patient’s tumor, the uncertainty in data collection, and the desirable error range for model prediction. Though we demonstrated this in untreated breast cancer, we are confident that the methods described here can be expanded to other cancer types. We hope that this study can help inform future data collection for accurate prediction of patient-specific tumor growth dynamics.

## Data Availability

The data are available upon reasonable request.

## Supplementary Materials

The following supporting information can be downloaded at: https://www.mdpi.com/article/10.3390/cancers15051368/s1, Figure S1: Distribution of Patient-Specific Carrying Capacities; Figure S2: Error distributions of model fit to data with 10% noise; Figure S3: Error distributions of model fit to data with 20% noise.
