# Supplementary material for: Predicting Patient-Specific Tumor Dynamics: How Many Measurements Are Necessary?
Source: Cancers (Basel). 2023 Feb 21;15(5):1368. doi: 10.3390/cancers15051368 (PMC10000065; doi:10.3390/cancers15051368)
Supplement: Supplementary file 1 [file cancers-15-01368-s001.zip › cancers-2183997-supplementary.pdf]

Supplementary Figures

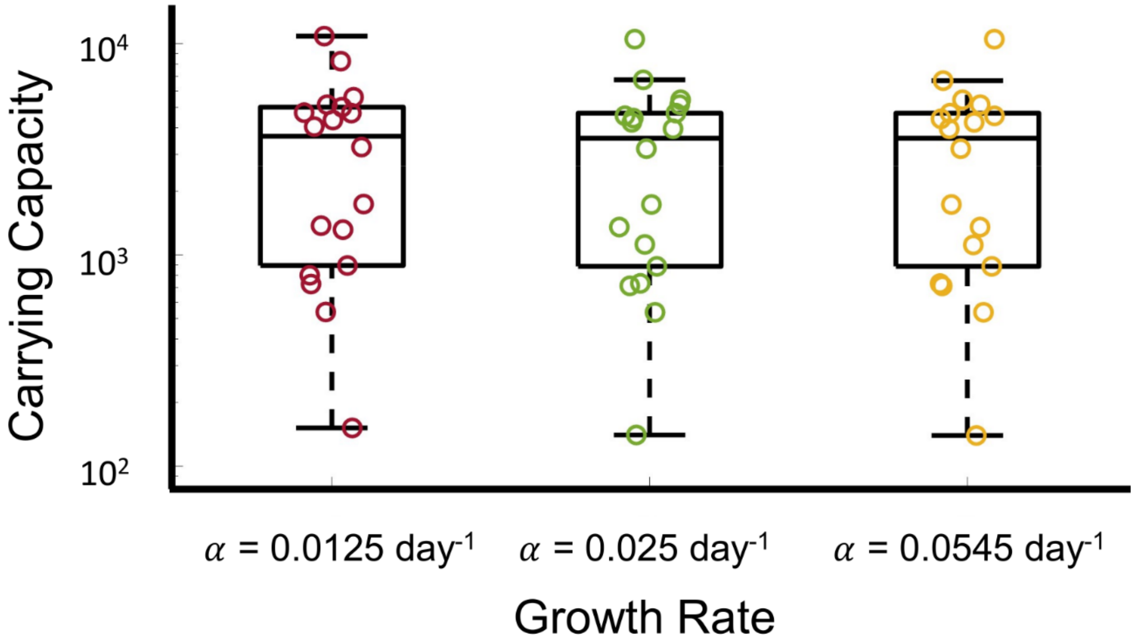

Figure S1. Distribution of Patient-Specific Carrying Capacities. These are the original patient-specific carrying capacities found using parameter optimization at α=0.0125 day^{-1}, 0.025 day^{-1}, and 0.0545 day^{-1}. These carrying capacities served as the "ground truth" to which later model parameters were compared to for error analysis.

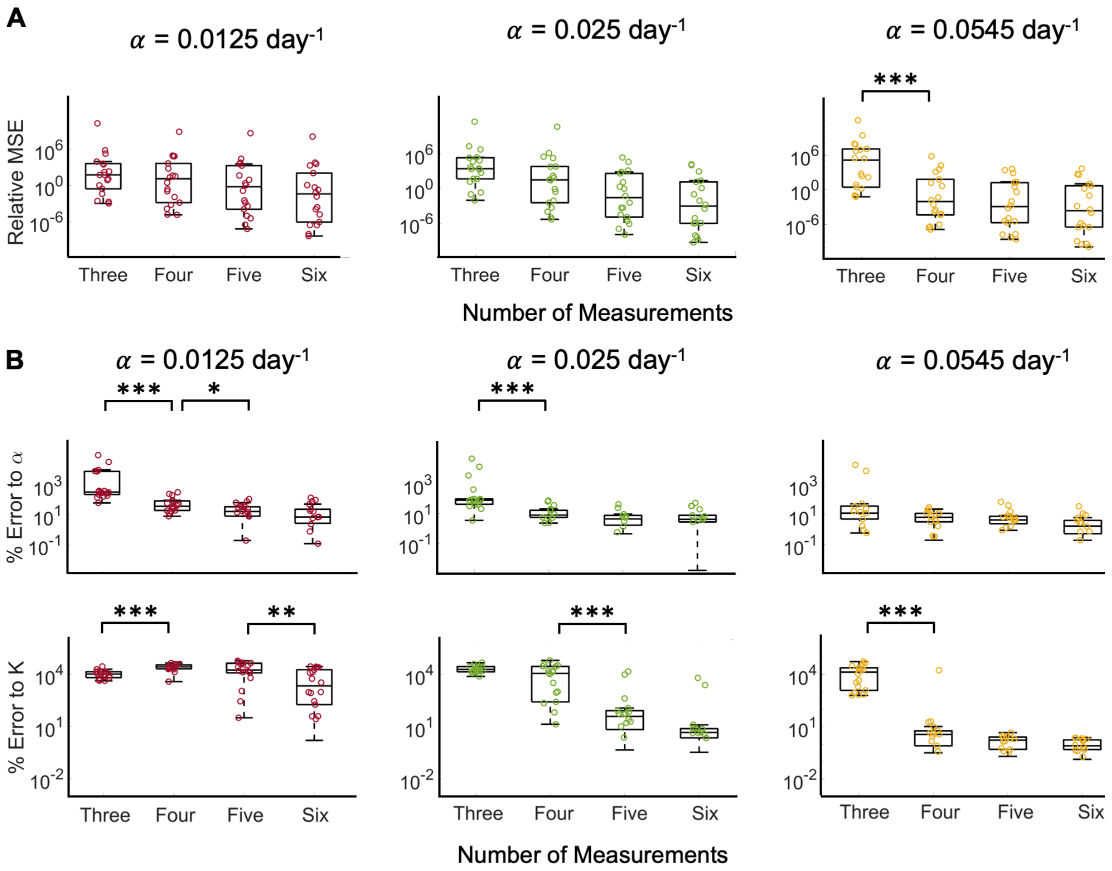

Figure S2. Error distributions of model fit to data with 10% noise. A. Relative mean square error (MSE) of model fit to ground truth data with different growth rates as a function of different measurements included in parameter estimation. **B.** Percent error of model fit-derived logistic growth parameters (growth rate, α, and carrying capacity, K) to ground truth parameters as a function of different measurements included in parameter estimation.

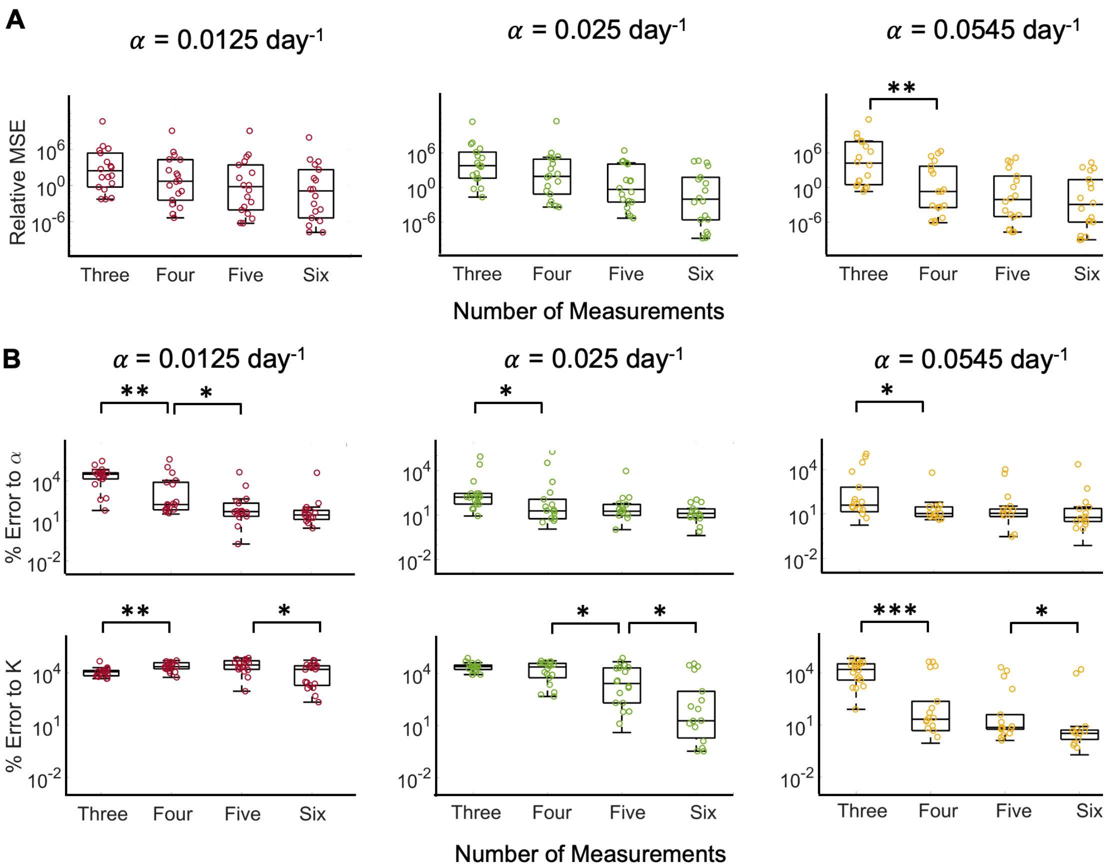

Figure S3. Error distributions of model fit to data with 20% noise. A. Relative mean square error (MSE) of model fit to ground truth data with different growth rates as a function of different measurements included in parameter estimation. **B.** Percent error of model fit-derived logistic growth parameters (growth rate, α, and carrying capacity, K) to ground truth parameters as a function of different measurements included in parameter estimation.
